# Sol–Gel Technologies to Obtain Advanced Bioceramics for Dental Therapeutics

**DOI:** 10.3390/molecules28196967

**Published:** 2023-10-07

**Authors:** Xiaozhe Song, Juan J. Segura-Egea, Aránzazu Díaz-Cuenca

**Affiliations:** 1 Materials Science Institute of Seville (ICMS), Joint CSIC-University of Seville Center, 41092 Sevilla, Spain; xiaozhe.song@icmse.csic.es; 2 Department of Stomatology, Faculty of Dentistry, University of Seville, 41009 Seville, Spain; segurajj@us.es

**Keywords:** bioactive glasses, calcium phosphates, calcium silicates, bioceramics, regenerative dentistry

## Abstract

The aim of this work is to review the application of bioceramic materials in the context of current regenerative dentistry therapies, focusing on the latest advances in the synthesis of advanced materials using the sol–gel methodology. Chemical synthesis, processing and therapeutic possibilities are discussed in a structured way, according to the three main types of ceramic materials used in regenerative dentistry: bioactive glasses and glass ceramics, calcium phosphates and calcium silicates. The morphology and chemical composition of these bioceramics play a crucial role in their biological properties and effectiveness in dental therapeutics. The goal is to understand their chemical, surface, mechanical and biological properties better and develop strategies to control their pore structure, shape, size and compositions. Over the past decades, bioceramic materials have provided excellent results in a wide variety of clinical applications related to hard tissue repair and regeneration. Characteristics, such as their similarity to the chemical composition of the mineral phase of bones and teeth, as well as the possibilities offered by the advances in nanotechnology, are driving the development of new biomimetic materials that are required in regenerative dentistry. The sol–gel technique is a method for producing synthetic bioceramics with high purity and homogeneity at the molecular scale and to control the surfaces, interfaces and porosity at the nanometric scale. The intrinsic nanoporosity of materials produced by the sol–gel technique correlates with the high specific surface area, reactivity and bioactivity of advanced bioceramics.

## 1. Introduction

Oral diseases remain the most dominant conditions globally [1]. Overall, the estimated number of cases of oral diseases is about 1 billion times higher than the cases for all five of the main noncommunicable diseases (NCDs) combined: mental disorders, cardiovascular disease, diabetes mellitus, chronic respiratory diseases and cancers [2]. Untreated caries in permanent teeth are the most prevalent, which is followed by severe periodontal disease, and then untreated caries in deciduous teeth and edentulism [2]. In addition, one of the most interesting aspects currently facing dentistry is the possible connection between chronic oral inflammatory processes of infectious origin (chronic apical periodontitis and periodontal disease) and systemic health status [1,3,4]. Several epidemiological studies highlight the connection between chronic oral inflammation and systemic diseases, such as ischemic heart disease [5], hypertension [6], diabetes [7], metabolic syndrome [8], renal disease [9,10], inflammatory bowel disease [11,12], rheumatoid arthritis [13], osteoporosis [14], memory loss [15], adverse pregnancy outcomes [16], cancer [17,18], respiratory diseases [19] and COVID-19 [20].

To improve the oral and dental health of the population, regenerative dentistry is a very promising approach that aims both to prevent oral–dental deterioration and to restore the anatomy and functionality of diseased teeth [21]. To this end, it draws on new advances in procedures based on cell biology and new biomaterials [22,23]. Synthetic biomimetic materials, and particularly bioceramics are undoubtedly fundamental elements in the development of these advanced dental therapies, as the calcium phosphate type formulations are the natural bioceramic components of dental and bone tissues [24,25]. To this must be added the breakthrough achieved with the new silica-based bioactive ceramic compositions, capable of stimulating biological mineralization processes [26,27].

Initially, and still in use in most of the products that are currently applied in the clinic, bioceramic materials have been synthesized from precursor salts using traditional industrial processes that required high temperatures, followed by the casting of bulk implants or the quenching of powders. However, since the early 1990s, research has begun on bioactive ceramics using an alternative process, the sol–gel technique [28,29]. The synthesis of nanomaterials can be broadly classified into two approaches: “top-down” and “bottom up”. Top-down synthesis involves the deconstruction of larger materials to produce nanostructures. Bottom-up synthesis constructs nanomaterials from basic building blocks like atoms and molecules. The sol–gel technique is an example of a bottom-up approach for producing bioceramics from small molecules. The method consists of several stages involving chemical and physical processes. The chemical process begins with the reaction of precursor monomers to form oligomers in solution (sol), which in turn polymerize into a network (gel) in the form of an integrated network of discrete particles or network polymers [30]. In general, the mechanism of hydrolysis of the precursor monomers and their condensation to oligomers are the most critical steps in sol–gel chemical synthesis. These mechanisms determine the structure and composition of the resulting material. The synthesis parameters that bias the structure toward linear or branched structures are also critical issues, which play a crucial role in determining the properties and performance of the final material.

A major advantage of the sol–gel method is that it is possible to obtain materials with high purity and homogeneity at the molecular scale and to control the surfaces, interfaces and porosity of the materials obtained at the nanometric scale [31]. The sol–gel method produces homogeneous sols that can be converted into gels with a very high volume of nanopores. This nanoporosity is undoubtedly one of the most important characteristics of advanced bioceramics, as it translates into a higher specific surface area, greater reactivity and, therefore, faster kinetics in the bioactive response [32]. The sol–gel method can be used to synthesize bioceramics with different chemical compositions, which enables the production of a wide range of materials. Besides, it is a cost-effective technique, as it requires lower reaction temperatures and simpler equipment than other high temperature burning and thermal industrial processes. The low reaction temperature reduces the energy consumption and gas emissions, which contribute to its environmental sustainability.

On the other hand, control of the processing method makes it possible to vary the morphology of the synthesized materials and, thus, obtain particles, films, monoliths or fibers [33,34]. Additionally, the whole process can be easily scaled up for large-scale production. All these characteristics make it possible to obtain bioceramics with high added value, and there is much interest in finding new synthesis routes and processes that align these advantages with other equally important commercial aspects, with respect to the economic viability of scaling up sol–gel production [31].

The aim of this work is to review the application of bioceramic materials in the context of current regenerative dentistry therapies, focusing on the latest advances in the synthesis of advanced materials using the sol–gel methodology. Chemical synthesis strategies and important parameters, and the processing and therapeutic possibilities are discussed for the three most relevant types of bioceramic-based materials: bioactive glasses and glass ceramics, calcium phosphates and calcium silicates.

## 2. Commercial Bioceramics Currently in Use

**Figure 1:**
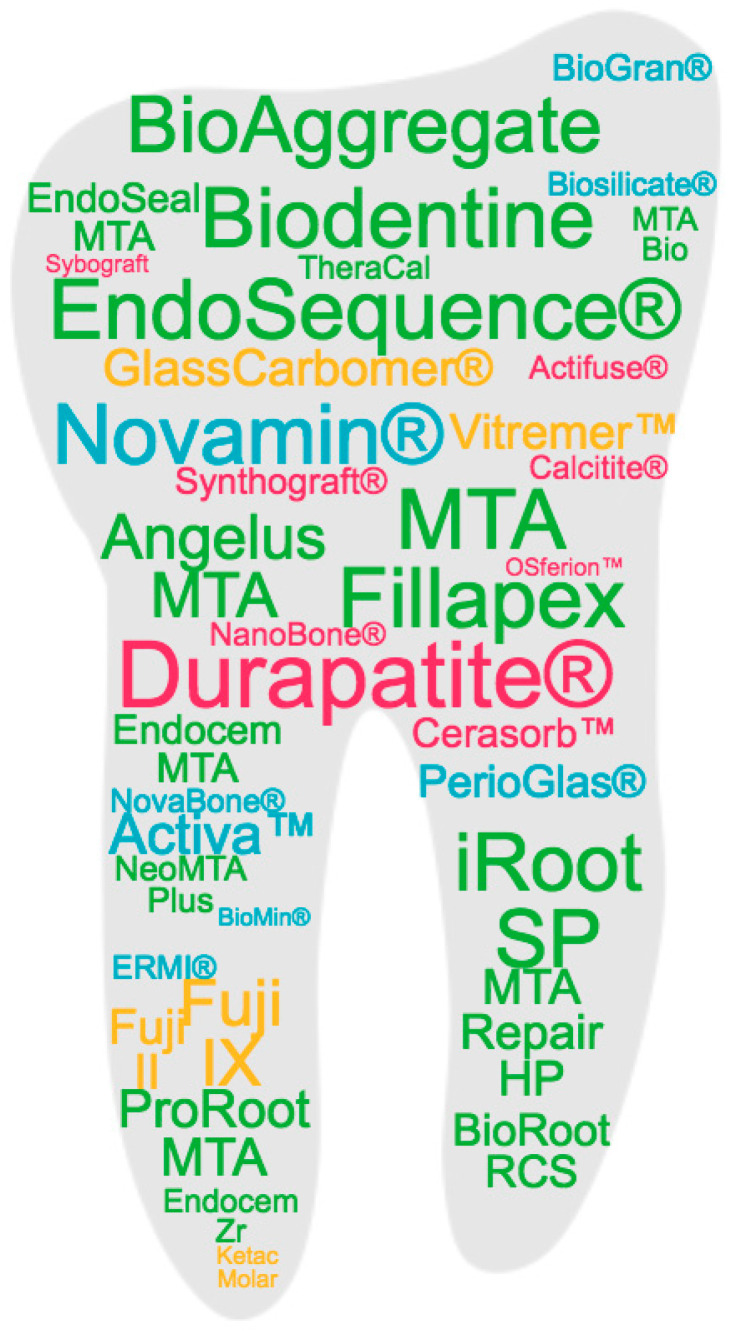
Commercial products based on bioceramics in clinical use that are most studied in the literature. Based on a search of the Scopus database, the products are represented with a font size according to their frequency in the title of an article and with a color code corresponding to the different types of bioceramics classified in the work: blue for bioactive glass (BG); yellow for glass ionomeric; pink for calcium phosphate (CaP); green for calcium silicate (CaSi).

There are a large number of bioceramic materials on the market that are used in dentistry to stimulate the repair and regeneration of dental tissues, such as enamel, dentin and pulp, as well as bone defects in oral and maxillofacial surgeries. Most of them correspond to ceramic products obtained by conventional methods, such as melting in the case of vitreous materials or high temperature heat treatment of precursor salts. There are, however, a few commercially available products, such as NovaBone or NanoBone, that have begun to incorporate advances in sol–gel synthesis, giving them new textural properties in terms of surface and porosity. Figure 1 represents the commercial products based on bioceramics in clinical use that are most studied in the literature, marked in different colors according to the main type of bioceramic material component, namely bioactive glass (BG), calcium phosphate (CaP) or calcium silicate (CaSi), as detailed in the following subsections.

### 2.1. Bioactive Glasses

Bioactive glasses (BGs) were discovered by Hench in 1969 [35]. His team at the University of Florida found that these materials elicited a biological response when they came into contact with the physiological environment, which led to a new approach for the application of biomaterials in clinical practice [36,37,38]. The original bioactive glass composition, 45S5, formulated on weight bases from 45% SiO_2_, 24.5% Na_2_O, 24.5% CaO and 6% P_2_O_5_, was commercially trademarked as Bioglass^®^ [35] and many of the commercial products still available use this composition. Variations in this formulation, including other compounds such as K_2_O, MgO, CaF_2_ and B_2_O_3_, have been implemented and have shown altered properties such as dissolution rates and bioactivity. In addition to variations in their composition, different processing methods have also been reformed, such as their manufacture in the form of implant-like monoliths, granules or particles, pastes and cements. An excellent recent paper reviews all of the commercial BGs devices approved for therapeutic application, including hard tissue scaffolding, dental remineralization, soft tissue repair and cancer treatment [39].

In the field of dentistry, Endosseous Ridge Maintenance Implant (ERMI^®^), PerioGlas^®^, BioGran^®^, NovaBone^®^ and NovaMin^®^ are commercialized bioactive glass products based on the 45S5 composition. ERMI^®^ is used in the form of a monolith to be implanted into the void left following tooth extraction to encourage bone formation and to provide a stable ridge for future tooth replacement [40,41,42]. PerioGlas^®^ is used for the repair of periodontal and smaller oral defects. It was the first product to be delivered as glass powder, ranging from 90 to 710 μm, which makes surgery easier by allowing the operator to pack the wound with powder rather than fit a premade product into the void [43,44,45]. BioGran^®^ has a similar application to PerioGlas^®^, but with a narrow particle size of 300 to 360 μm [46,47,48,49]. NovaBone^®^ is available in the form of dental putty combined with a binder to improve handling for grafting, and also as interconnected porous granules for faster bone integration and remodeling for reconstructive surgeries, such as ridge maintenance and augmentation, extraction sites, implant preparation and placement [50,51,52]. NovaMin^®^ is applied to toothpaste for treating tooth hypersensitivity. It has a fine particle size with a D50 of 18 μm, which allows the glass to have a higher probability of entering the dentin tubules in the teeth [53,54,55,56,57,58]. Besides, NovaMin^®^ is used in polishing and teeth whitening procedures to stimulate mineralization [33] and has been shown to help to treat gingivitis [59]. BioMin^®^ is a modification of the 45S5 composition containing either fluorine (BioMin F) or chlorine (BioMin C) to aid in apatite precipitation for dentin hypersensitivity [60,61]. Another glass composition variation (wt.%), 48.5% SiO_2_, 23.75% Na_2_O, 23.75% CaO and 4% P_2_O_5_, is used to produce the Biosilicate^®^ glass ceramic. Biosilicate, engineered under a controlled double-stage heat treatment, is effective in the clinical reduction of sensitivity in enamel and dentine [62,63,64].

Bioactive glasses of undisclosed exact composition are also marketed as components in composite formulations with resins, polymers and other agents for use as restorative esthetic composites and biomaterials for endodontics. Activa™ BioACTIVE contains a shock-absorbing component, making it resistant to fracture and wear. It chemically bonds to the tooth and releases and recharges calcium, phosphate and fluoride ions, providing long-term benefits [65,66]. GuttaFlow^®^ is composed of gutta-percha, polydimethylsiloxane, platinum catalyzer, zirconium dioxide and BG, showing low solubility, low porosity, alkalization capacity, dentin penetrability and cytocompatibility [67]. A newly developed bioactive glass-based cement, Nishita Canal Sealer BG (NCS-BG), is now being commercially marketed as a root canal sealer and applied within clinical endodontic treatments [68].

Finally, Ting et al. [69] reported that the 58S glass (nominal composition 60 mol% SiO_2_, 36 mol% CaO and 4 mol% P_2_O_5_) was one of the first sol–gel-derived bioactive glass compositions developed and commercialized by NovaBone Products LLC (Alachua, FL, USA), although hydroxyapatite (HA) was found to form within the 58S glass during sol–gel synthesis after thermal stabilization, where it was heated to 700 °C.

Although with a distinctive bioactive ability in restorative dentistry, glass-ionomer materials also deserve a separate mention. They are a group of materials composed of silicate glass powder and an aqueous solution containing polyacrylic acid that solidifies due to an acid–base reaction [21]. Glass-ionomer cements (GICs) are considered bioactive because they release biologically active ions, such as fluoride, calcium, strontium, sodium, phosphate or silicon, that result in long-term durable bonds at the tooth–restoration interface [70,71]. Commercial GICs, Fuji IX [72,73,74,75], Ketac Molar [76,77], Glass Carbomer^®^ [78,79,80,81], have been shown to promote remineralization in the mouth. Resin-modified products, Fuji II [82,83,84] and Vitremer™ [85,86,87], also contain ion-leachable glass powder, as well as the water-soluble organic monomer 2-hydroxyethyl methacrylate (HEMA), and are widely used as alternatives to amalgam.

### 2.2. Calcium Phosphates

Synthetic calcium ortho-phosphate (CaP) materials can be prepared with a chemical composition very similar to that of the inorganic part of human bones and teeth. They are widely used in medicine for their biocompatibility, bioactivity and osteoconductivity properties [88]. Bone and dentine contain about 70% calcium phosphate (CaP) mineral in the form of a poorly crystalline, highly substitute apatite phase, consisting of very small crystallites, with a thickness of only 5 nm. Enamel, on the other hand, consists almost exclusively of hydroxyapatite prisms up to 100 µm in length and oriented in structures that confer resistance to abrasion [24]. Several dental specialties deal with the invasion into or the treatment of the surrounding bones, such as the filling and/or reconstruction of a traumatic or degenerative multi-walled bone defect, augmentation of the sinus floor, augmentation of alveolar ridges, the filling of periodontal or other alveolar bone defects, tooth sockets, osteotomies and the preservation of the alveolus for the preparation of an implant site. Depending on the application, different compounds, such as monocalcium phosphate (MCP; Ca(H_2_PO_4_)_2_), dicalcium phosphate (DCPA; CaHPO_4_), tricalcium phosphate (TCP; Ca_3_(PO_4_)_2_) or hydroxyapatite (HA; Ca_10_(PO_4_)_6_(OH)_2_), as well as their processing in different formats, such as particles, granulates, dense blocks, porous parts, pastes or coatings, have been developed. Research on this type of material is very extensive, as some recent reviews in the bibliography show [89,90,91]. Dorozhkin [89] highlights that the first publications on the application of CaPs in dentistry deal with their inclusion in toothpaste formulations to promote remineralization and reduce tooth sensitivity, and reviews these materials according to two types of classification, namely the CaP compound formulation and the specific application for the different specialties in dentistry [89].

The first reported commercial CaP products are based on β-TCP and HA, such as Synthograft^®^ (β-TCP) [92,93,94,95], Durapatite^®^ (HA) [96,97,98,99], Calcitite^®^ (HA) [100,101] and Alveograft^®^ (HA) [102]. Also, β-TCP based products are subsequent to Cerasorb™ [103,104,105] and OSferion™ [106]. Actifuse^®^ is a porous silicate-substituted calcium phosphate [107,108]. Synthetic nano-crystalline HA is commercialized as a single component bone graft by Sybograft^®^ [109], and as a composite formulation NanoBone^®^ consisting of nanocrystalline HA embedded in a silica gel matrix, produced using a sol–gel process [110,111,112]. Besides, CaPs are incorporated as components in self-setting products, such as Endo Sequence^®^ BD Sealer, a premixed ready-to-use injectable cement for sealing applications, which contains MCP [89,113].

### 2.3. Calcium Silicates

Calcium silicates, mainly Ca_3_SiO_5_ and Ca_2_SiO_4_, are the basic compounds in bioactive endodontic cements (BECs) [26,114]. BECs are bioceramics widely used in endodontics as restorative cements used in vital pulp therapy and endodontic sealers, due to their high biocompatibility, intrinsic osteoconductive activity and ability to induce regenerative responses as dentin bridges that promote better sealing of the pulp-capped site [66,115]. These calcium silicates compounds are capable of reacting with water at a physiological temperature, causing a hydraulic setting reaction. Originally the first product formulation was described as a powder composed of calcia, silica and alumina oxides and was then named mineral trioxide aggregate (MTA), which is still a generic name used for BECs in dentistry [116]. In fact, MTA is based on Portland cement, which was revisited by Torabinejad et al. [117] for its use in endodontics. Despite its excellent properties, some problems in its clinical application, such as the long setting time, tooth discoloration, high cost and difficult handling, have driven the development of new formulations.

The first clinically approved formulation was ProRoot MTA [118]. The initial setting time has been reported from 70 to 74 min [114]. In 2002, the gray ProRoot MTA (GMTA) was substituted by the new white ProRoot MTA (WMTA), free from tetracalcium aluminoferrite to reduce the problems concerning tooth discoloration [119]. MTA Angelus [120,121] followed with a similar composition based on Portland cement, but without the calcium sulphate dehydrate (gypsum). Further products marketed with shorter setting times are Biodentine [122,123,124,125,126,127,128,129], Endocem MTA [130,131,132,133], MTA Bio [134,135], EndoSeal MTA [136,137] and MTA Fillapex [138,139,140,141,142,143]. A setting time of as little as 0.3 min has been reported for TheraCal [144,145,146,147,148] because of the use of resin and light cure technology. The radiopacifying agent used is another important element that has been studied for the improvement of these products. ProRoot MTA contains about 2 at.% Bi [119], which may not only produce tooth discoloration but also reduce its biocompatibility [149]. As an alternative to bismuth oxide, other compounds have been used, such as tantalum oxide Ta_2_O_5_, which is used in BioAggregate [150,151,152,153,154] and NeoMTA Plus [26,155,156,157]. ZrO_2_ is another agent widely used in products such as Endocem Zr [131,158], EndoSequence [159,160], iRoot SP [161,162,163,164], BioRoot RCS [165,166,167,168] and the previously mentioned Biodentine. MTA Repair HP, notable for its low setting time and fast bioactive response in vitro [25], contains CaWO_4_ as a radiopacifying agent and consists of tricalcium silicate nanoparticles with high aspect ratio, which provide to the precursor material an elevated surface area to maximize the hydration reaction [169,170].

## 3. Current Research on Sol–Gel Bioceramics for Application in Dentistry

The chemistry of the sol–gel technique offers great versatility and can be used for the preparation of a wide variety of bioceramic compositions and different macro-, micro- and nanostructure features for application in regenerative dentistry.

### 3.1. Basics of the Sol–Gel Synthesis Technique

The sol–gel method is a wet-chemistry process, which involves several stages from the initial precursors solution (sol) to the gelation (gel) phase. The interest in and development of this process dates back more than 150 years, when it was discovered that hydrolysis under acidic conditions of the compound tetraethyl orthosilicate (TEOS) produced SiO_2_ in the form of a glass-like material [30,171]. A typical process starts with the hydrolysis and polycondensation reactions of the precursor alkoxide-type compounds. Silicon alkoxides represent the main network forming agents in sol–gel preparation methods and tetraethyl orthosilicate (TEOS) is still the most widely used silicate precursor, while water and/or ethanol are used as solvents. The formation of the silicate network follows a widely accepted two-stage process [30,172]: hydrolysis (Equation (1)) and condensation (Equations (2) and (3)). Hydrolysis and condensation may occur simultaneously as silanol groups on partially hydrolyzed molecules that undergo condensation:

Si(OR)_4_ + 4 H_2_O → Si(OH)_4_ + 4 ROH (1)

2 Si(OH)_4_ → (OH)_3_Si-O-Si(OH)_3_ + H_2_O (2)

Si(OR)_4_ + Si(OH)_4_ → (OH)_3_Si-O-Si(OR)_3_ + ROH (3)

By modifying the synthesis parameters, the properties of the final materials, such as the morphology and composition, can be controlled and designed. In general, the rate of hydrolysis is fast compared to that of condensation in strong acid conditions and, alternatively, a higher pH favors condensation. However, a higher and lower pH is able to promote condensation and hydrolysis and, in silica based systems, the reactions proceed as a result of acid catalysis at a pH < 2 and basic catalysis when the pH > 2, around the isoelectric point of silica at pH = 2 [173]. Silicon alkoxides tend to form a 3D gelled structure under acid conditions or individual particles under basic conditions [31,174]. The controlled growth of monodisperse silica spheres was first achieved by Stöber et al. [175], using a base-catalyzed sol–gel synthesis route involving silica alkoxide precursors and an ammonium hydroxide catalyst. Moreover, using appropriate template molecules a well-ordered hexagonal arrangement of mesopores is formed at low pH acid conditions [176].

Other silane oligomers capable of taking part in the hydrolysis and condensation reactions can be also used, apart from TEOS [177]. Besides, network modifier elements, such as calcium or magnesium, can be introduced in the form of inorganic salt or as alkoxide precursors [28,178,179]. Like silicon, phosphorous can be used a network former within the sol–gel process [28]. However, the σ-π double bond reduces the expected coordination number and produces a more relaxed network structure when compared to silica-based materials [31]. Besides, the low reactivity to acid-catalyzed hydrolysis of triethyl phosphate (TEP) should be noted, which is the most commonly used phosphorous precursor. Studies have shown a large loss of phosphorous for the TEP-prepared gels, most likely due to the much lower rate of hydrolysis of TEP than the silica precursor TEOS [180,181].

**Figure 2:**
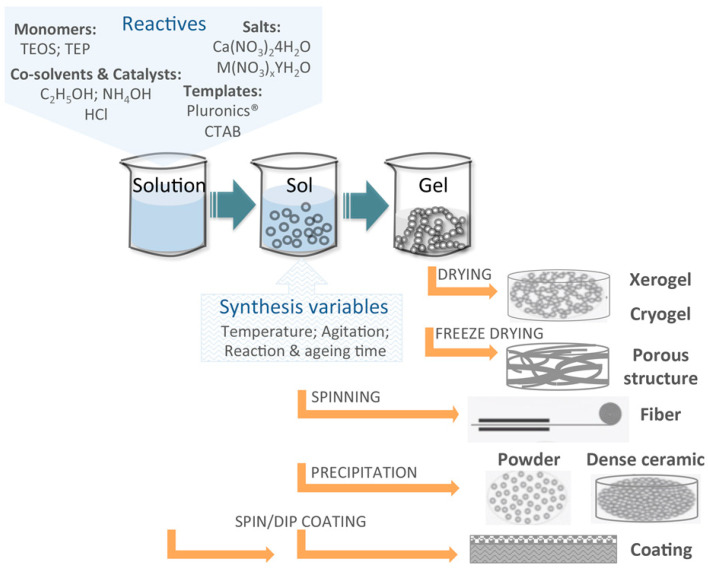
Chemistry stages and processing strategies of sol–gel technologies.

In short, by varying the different synthesis variables in the process, such as the silicon or phosphorus monomers, the salt precursors, the use of template molecules, co-solvents, pH catalysts, the temperature and reaction times, as well as the different post synthesis treatments and processing routes, a large number of formulations in different useful morphologies and formats can be produced (Figure 2).

Although the synthesis of bioceramics using the sol–gel technique is considered safe, there are some health and safety hazards that must be considered. Thus, as mentioned, the synthesis involves the use of alkoxides, acids and solvents, which might require appropriate safety protocols, such as wearing protective clothing and gloves, working in a well-ventilated area and using fume hoods when necessary. Some sol–gel precursors and reaction by-products may release toxic fumes during the synthesis process. Besides, the sol–gel process may generate fine particles or dust, which can pose a respiratory hazard if inhaled. It is, therefore, advisable to wear appropriate respiratory protection equipment, such as masks or respirators.

### 3.2. Progress in Bioactive Glasses Research

#### 3.2.1. Compositions and Chemical Routes

The original 45S5 composition, 45%SiO_2_—24.5%Na_2_O—24.5%CaO—6%P_2_O_5_ [36], was obtained by a process of melting the precursor salts, followed by the casting of the bulk implants or cooling to get a material in particulate form. This conventional melt processing has a major limitation in terms of the compositional variability, as it must be limited to phase diagram formulations that are within the glass-forming region. In 1991, the first stable bioactive-gel glass could be made by sol–gel processing without sodium from the composition [28], reducing the glass from a four- to a three-component system. The material was prepared from TEOS, TEP, calcium nitrate, Ca(NO_3_)_2_4H_2_O (CaN) and nitric acid to accelerate the hydrolysis of TEOS. After mixing the components, the sol was gelled, aged and dried at 60–180 °C [28]. Finally, the dried gels were heated at 600–700 °C, which allows the glassy materials to be obtained using heat treatment of the gels at more moderate temperatures than required for melting. A series of compositions within the SiO_2_-CaO-P_2_O_5_ system were further studied, and in vitro bioactivity in simulated body fluid (SBF) was demonstrated for sol–gel glass compositions with nearly 90% SiO_2_. In fact, the rate of surface biomimetic hydroxy carbonate apatite (HCA) formation for the 60%SiO_2_-36%CaO-4%P_2_O_5_, named the 58S composition, was even more rapid than from the melt-derived 45S Bioglass^®^ [28]. The first suggested explanations for these good results were the presence of nanopores and, consequently, their high specific surface area, above 200 m^2^ g^−1^ for these sol–gel materials. These textural characteristics were related to an increase in the density of potential sites for the nucleation and growth in the superficial hydroxyapatite layer [182].

Going back to the pioneering sol–gel formulations by Li et al. [28], all synthesized bioactive compositions were not fully glassy, as they contained some crystalline phases. Hence, to produce a completely amorphous 45S5 BG composition without crystalline inclusions using the sol–gel technique has been a challenge so far and several authors have reported the presence of calcium sodium silicate phases [183,184,185,186]. Faure et al. [183] explored the use of citric acid instead of the usual nitric acid for the synthesis of the 45S5 formulation, but revealed a partial crystallization of Na_2_Ca_2_Si_3_O_9_, Na_2_Ca_3_Si_6_O_16_ and Na_2_CaSi_2_O_6_ inside the amorphous structure of the BG. However, Esfahanizadeh et al. [187] showed that zinc-doped BG had a much lower crystalline phase compared to 45S5 BG, and Shankhawar et al. [188] demonstrated that when using (NH_4_)_2_HPO_4_ as the phosphate precursor, it is possible to obtain fully amorphous material with a composition close to 45S5 BG.

Vallet-Regí’s group used the sol–gel route for the preparation of bioactive glasses in the ternary SiO_2_-CaO-P_2_O_5_, quaternary SiO_2_-CaO-P_2_O_5_-MgO and the binary SiO_2_-CaO systems [189]. The results obtained for the ternary system compositions using SiO_2_ content from 55 to 80 mol%, indicated significant variations in the textural properties, such as the pore size, pore shape and specific surface area in relation to the relative proportions of the three components. Besides, the addition of MgO to obtain quaternary glasses was performed to investigate the role of magnesium to improve the mechanical features of the glasses, while the binary SiO_2_-CaO compositions were studied to determine the role of phosphorous in the glasses’ bioactivity. The ternary system, SiO_2_-CaO-P_2_O_5_, is perhaps one of the most studied and, particularly, the formulation referred to in the literature is 58S. This formulation can be found in several works, in a narrow range of compositions expressed in % molar for SiO_2_ (58–60), CaO (36–38) and P_2_O_5_ (4) [28,182,189,190]. The effect of using ethanol and ammonia solution in 58S glass synthesis for dental applications has recently been studied and found to produce small glassy grains and more porous surfaces [190].

Perhaps one of the most interesting contributions of Prof. Hench’s work on BGs, is the finding that Ca and Si ionic dissolution products released from BG stimulate the genes of cells towards a path of regeneration and self-repair [191]. Closely related to this, there is growing evidence in the literature that the dissolution products from other chemical elements, such as Zn, F, Sr or Cu, can help enhance the biological response to assist tissue regeneration processes [192]. In this sense, it is worth highlighting the great versatility of sol–gel chemistry to extend the composition of BG with other elements that can produce ionic dissolution products with therapeutic functionality. Fluoride-containing sol–gel BGs have the potential to release F, Ca and PO_4_ ions promoting remineralization. F-BG (~5% mol.%) synthesis routes based on the 45S5 [193,194], 60S [195] and 77S [196] formulations have been tested using, respectively, NaF, CaF or HF reagents. The bactericidal properties of the elements Zn, Cu, Sr, Ag, Mg and Li have been exploited for their incorporation in sol–gel synthesis by means of nitrate precursor salts for the formulations 45S5 [186,187], 58S [197] and 50S (50 M% SiO_2_) [198]. The search for improvement in the mechanical properties of 45S5 and 58S sol–gel synthesis has been studied with the incorporation of Al(NO_3_)_3_9H_2_O [199] and ZrO_2_ [200] precursors, respectively. Also, to improve the micro-hardness of enamel, sol–gel synthesis of 70STi-modified glass (71.4 wt% SiO_2_–23.6 wt% CaO–5 wt% TiO_2_) has been carried out using titanium isopropoxide [201].

But a major breakthrough in the development of these materials has undoubtedly been the achievement of multicomponent bioactive glasses with ordered mesopores, known as mesoporous bioactive glasses (MBG) or “glasses obtained by template or structure-directing molecules”. MBGs are nanostructured bioceramics that are ordered at the mesoscale yet disordered at the atomic scale. They have been engineered in formulations of the ternary systems (SiO_2_-CaO-P_2_O_5_) and the simplest binary systems (SiO_2_-CaO), but also for the more complex extended compositions including other elements (such as Sr, Cu, Co, Zn, Mg, etc.). In these bioceramics, the material is distributed on the walls separating channel-shaped pores, which are arranged periodically in highly ordered structures. This ordered arrangement of mesopores, in the range between 2 and 50 nm and with uniform distribution, produces materials with textural properties (surface area and pore volume) approximately double those of conventional sol–gel BGs [202]. The 58S BG formulation has been synthesized in the form of mesoporous nanoparticles, with sizes in the 300–500 nm range, using dodecylamine (DDA) [203] or hexadecyltrimethyl ammonium bromide (CTAB) [204] as template molecules. Also, using CTAB, a variation of the ternary 60SiO_2_-30.8CaO-9.2P_2_O_5_ composition with the incorporation nitrogen was achieved using a different amount of ethylenediamine, C_2_H_8_N_2_ as a nitrogen source [205]. MBG nanoparticles of 85SiO_2_-15CaO (mol%) have been synthesized using CTAB as a template and aqueous ammonia as a catalyst and a different combination of reactives, including ethanol and 2-ethoxyethanol [206] or poly(ethylene glycol) (PEG) [207]. Expanded boron-containing MBGs, based on (60-x)SiO_2_-xB_2_O_3_-30.2CaO-9.8P_2_O_5_ (x = 0, 5, 10, 20 mol.%), using a CTAB template [208] and Sr-MBG nanoparticles of 85Si:10Ca:5Sr, were synthesized by the ultrasound-assisted sol–gel method (alkali-mediated), using PEG as a structural template [209]. The synthesis of combined supramolecular chemistry using block copolymers as structure directing agents, with evaporation-induced self-assembly (EISA), is another successful route for the preparation of MBGs. Pluronics^®^ are a class of commercial synthetic block copolymers, which consist of hydrophilic poly(ethylene oxide) (PEO) and hydrophobic poly(propylene oxide) (PPO), arranged in an A-B-A triblock structure. Silver-containing MBG, in which the mole percentages of Si, Ca and P are 80, 15 and 5 and to which 1 mol% Ag was added, has been achieved using Pluronic F-127 [210,211]. Cu-containing mesoporous bioactive glass (MBG) microparticles, with a Si:Ca:P:Cu molar ratio of 80:10:5:5, were successfully prepared using Pluronic P123 [212].

#### 3.2.2. Processing and Final Formatting of Materials

The first sol–gel compositions obtained in the SiO_2_-CaO-P_2_O_5_ ternary system were obtained in the form of particles of the order of 100–700 microns [28]. Spherical particles of 0.65 µm in size with the binary 30CaO-70SiO_2_ composition were obtained, after the introduction of the precursor sol into a tube furnace at 600 °C by an ultrasonic nebulizer [213]. The textural properties of high specific surface area and pore volume of the spherical mesoporous nanoparticles, as opposed to irregular microparticles, are being exploited for the manufacture of dental cements with good handling and setting times. Spherical, sub-micron bioactive glasses with final compositions close to 82%SiO_2_-15%CaO-3%P_2_O_5_ (M%), and rapid setting time properties of 10 min when mixed with PBS, have been achieved using dodecylamine (DDA) (serving as both a catalyst and template) [214]. Other interesting Sr-free and Sr-doped MBG of 85SiO_2_-15CaO and 85SiO_2_-10CaO-5SrO (wt%) have been successfully processed using a phosphate-buffered saline (powder to liquid ratio; P/L = 0.5 g mL^−1^) to form a soft cement paste that hardens within 5–10 min in the ambient environment [215].

**Figure 3:**
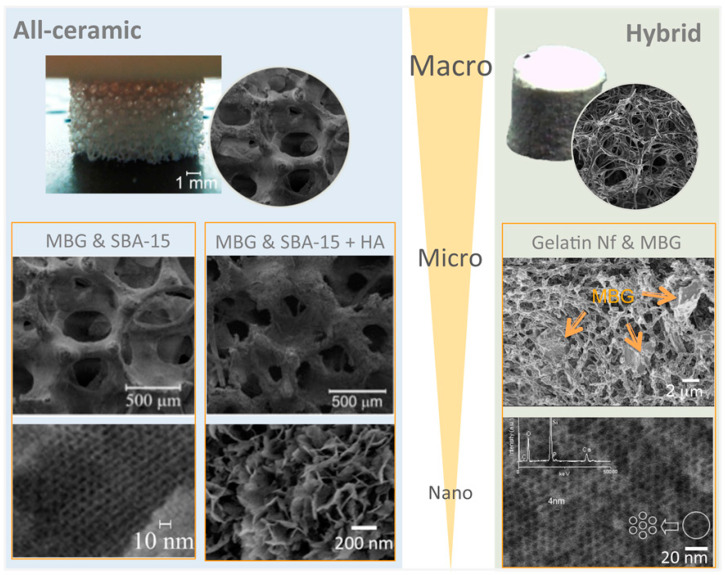
Bioceramics processed in reticulated structures with dual porosity in the micro and nanometer range: (**Left**) all ceramic structures composed of MBG and SBA-15 particles with and without a biomimetic HA coating; (**Right**) hybrid structure formed by stabilized gelatin nanofibers hosting microparticles of MBG. These bioceramics have been prepared in our laboratory at the ICMS. Further information on the preparation and characterization of such materials can be found in works 32, 34 and 235 in the reference list.

The ternary composition 60%SiO_2_-36%CaO-4%P_2_O_5_ (M%), a glass foam with a controlled macroporous structure, was successfully produced using sodium lauryl ether sulphate as a foaming agent [216]. Binary 30CaO-70SiO_2_ system discs, containing both a nanoporosity averaging at 9 nm and a macroporosity ranging 10–300 µm with a specific surface area of 130 m^2^ g^−1^, have been fabricated for pulp capping regenerative endodontics, which cast into molds the sol of tetramethylorthosilicate (TMOS), CaN, polyethylene oxide (PEO), acetic acid and hydrofluoric acid to catalyze gelation [217]. Li-MBG (Li/Ca/P/Si = 5/10/5/80, molar ratio) scaffolds with hierarchically large pores (300–500 µm) and well-ordered mesopores (5 nm), by incorporating Li ions into the scaffolds, were successfully prepared using a replica of polyurethane sponges and showed that this approach yielded scaffolds with a favorable composition, microstructure and mesopores properties for cell attachment, proliferation and cementogenic differentiation of human periodontal ligament-derived cells (hPDLCs) [218]. Also, by using a polyurethane foam as the sacrificial template for the replication method, reticulated ceramic scaffolds were performed using an 80Si15Ca5P molar ratio MBG sol and increasing amounts of SBA-15 type silica particles (SP) as a ceramic precursor [32]. Furthermore, related hybrid scaffolds incorporating a fibrillar collagen coating of less than 1 wt% collagen per scaffold, have allowed a significant increase in the compressive strength, while preserving a high surface area and nanopore accessibility, as well as promoting hydroxyapatite mineralization [219]. These latter structures were generated in our laboratory at the ICMS and are shown in Figure 3.

Hybrid chitosan-based guided tissue regeneration (GTR) membranes, incorporating a two component BG, CaO-SiO_2_ produced by the Stöber process, were prepared by solving casting using chitosan as the polymer matrix [220]. Also, a 3D printed tyramine-modified gelatin/silk fibroin/copper-doped 58S bioactive glass hybrid scaffold for rat bone defects was constructed. The molar composition of Cu was varied by up to 10% by substituting Ca, and the mechanism of the profound angiogenesis effect regulated by copper was explored in vivo [221]. A hybrid device consisting of MBG microparticles embodied in a nanofibrillar biodegradable matrix has been reported by our laboratory at the ICMS, using appropriate thermally induced phase separation (TIPS) processing variables of 5.4% (*wt/v*) gelatin with a 50/50 water/ethanol (*v/v*) ratio (see Figure 3). The device comprises high surface area MBG microparticles within a fibrous matrix of 170 nm average diameter nanofibers gelatin, forming a meshwork of 0.2–1.6 µm range voids. Relevant for its possible application in regenerative dentistry, gentamicin sulphate (GS) antibiotic high loading capacity and sustained release ability, as well as its in vitro bioactivity and osteoprogenitor cells biocompatibility, supports long-term antibacterial and bone growth stimulation properties [34].

#### 3.2.3. Therapeutic and Clinical Uses

Sol–gel bioactive glasses could be used in the treatment of two of the most prevalent oral diseases, caries and periodontitis, promoting the remineralization of teeth and killing the main pathogens. Moreover, 45S5 sol–gel BG doped with 5 wt% of Li (BGLi) presented a greater antibacterial behavior than BG against the *A. actinomycetemcomitans* strain associated with periodontitis, due to the presence of Li ions. Enamel lesion was partially remineralized in vitro, when both sol–gel bioactive glasses (BG and BGLi) were applied on its surface, with micro-hardness recoveries around 45% [186]. Bioactive glass foams, using the 60%SiO_2_-36%CaO-4%P_2_O_5_ (M%) composition, have been shown to be effective in vivo in maintaining the thickness of the alveolar ridge, and the addition of platelet-rich plasma (PRP) in association with the foams improve bone formation [216]. The enamel anti-demineralization effects of orthodontic resins containing 70 (M%) SiO_2_ mesoporous bioactive glass nanoparticles (MBN) doped with gallium have been investigated [222]. Anti-demineralization testing in vitro has demonstrated that the degree of enamel demineralization decreased as the GaMBN concentration increased, which indicates that resins containing 5% GaMBN may be viable orthodontic adhesives for preventing white spot lesions (WSLs). Sr-doped nano bioactive glass cements can be considered as multifunctional biomaterials with high bioactivity, excellent biodegradability, fast therapeutic ion release and high drug loading capability, which potentiates its application in dentin–pulp complex regeneration therapy [215]. The co-delivered Sr and phenamil using sol–gel processed Sr-doped MBG (85Si:10Ca:5Sr) nanoparticles, demonstrated significant stimulation of adult stem cell differentiation in vitro and osseous/dentinal regeneration in vivo, through bone morphogenetic protein signaling pathways [209]. The incorporation of Sr (2.5, 5 and 10 mol.%) into MBG scaffolds has significantly stimulated alkaline phosphatase (ALP) activity and osteogenesis/cementogenesis-related gene expression of PDLCs being a promising bioactive material for periodontal tissue-engineering applications [223].

Fluoride-containing sol–gel BG containing adhesives have the potential to release F, Ca and PO_4_ ions for a prolonged period even in a low pH environment, thus promoting remineralization to prevent the formation of “white spot lesions” (WSLs) in orthodontic treatments [193]. F-BG (5% mol.%) has the potential to be used in dentifrices, restorative materials and for other dental applications [194]. Silver-containing mesoporous bioactive glass MBG-Ag sealing combined with Er:yttrium–aluminum–garnet (YAG) laser irradiation on human demineralized dentin specimens has been proven in vitro as a durable treatment option for dentin hypersensitivity [210].

### 3.3. Progress in Calcium Phosphate Bioceramics Research

#### 3.3.1. Compositions and Chemical Routes

The inorganic constituent of teeth is a poorly crystalline and highly substituted apatite (hydroxyapatite; HA), consisting of very small crystallites in the nanometric range [90], and the sol–gel method is an excellent route to design advanced biomimetic calcium phosphate biomaterials. As described above, the sol–gel method is a wet synthesis and, in the first stage, calcium and phosphorous from various sources are dissolved in water and ethanol or other suitable solvents, such as 2-butanol or acetic acid. In many published works, calcium diethoxide (Ca(OEt)_2_) or calcium nitrate (Ca(NO_3_)_2_4H_2_O; CaN) is reacted with triethylphosphite (P(OC_2_H_5_)_3_) or triethylphosphate (PO(OC_2_H_5_)_3_; TEP), either in an aqueous or organic solution [224]. TEP remains relatively stable despite triethyl phosphite and, is often chosen, although it is reported that TEP has a relatively low reactivity for hydrolysis [225]. Alternatively, non-alkoxide processing is possible using other precursors, such as calcium nitrate, calcium acetate monohydrate (Ca(CH_3_COO)_2_H_2_O) or calcium chloride (CaCl_2_) as a source of calcium, and phosphoric pentoxide (P_2_O_5_), ammonium hydrogen phosphate ((NH_4_)_2_HPO_4_), phosphoric acid (H_3_PO_4_) or sodium phosphate (Na_3_PO_4_) as a source of phosphorous [224,225]. Generally, the time of ageing at an ambient temperature of the prepared solutions varied from 2 to 72 h, drying (~100–150 °C) and, finally, heat treatment at elevated temperatures (~300–900 °C) [224]. Besides, iron and strontium [226] nitrate precursor salts have been added to the synthesis solutions to incorporate Fe or Sr divalent ions, which successfully replace the Ca ions in the HA crystal lattice without distorting its native structure. Mesoporous hydroxyapatite nanoparticles, with mesopores of 6 nm in size and a specific surface area of 66 m^2^g^−1^, were achieved using CaN, diammonium hydrogen phosphate, ammonium hydroxide (NH_3_H_2_O) and stearic acid (CH_3_(CH_2_)_16_COOH; SA), a biocompatible medium chain fatty acid that would function as an organic modifier [227].

Also very interesting is the use of structure-directing molecules, such as block copolymers, using strategies that combine the preparation of HA in the form of nanometric particles by nucleation and growth on a mesoporous silica matrix [27]. The synthesis procedure consists of a first step in an acid solution for the preparation of the Ca-doped silica matrix, using TEOS, CaCl_2_ 2H_2_O and the block copolymer Pluronic^®^ 123, followed by a second step where the mesoporous material is dispersed in a (NH_4_)_2_HPO_4_ solution at pH = 9, which facilitates the nucleation and growth of HA nanoparticles decorating the nanoporous silica matrix [228]. Moreover, calcium phosphate glass systems with a molar ratio of 48CaO-45P_2_O_5_-5Na_2_O-2ZnO have been achieved starting from the preparation of alkyl phosphate (by dissolving P_2_O_5_ in anhydrous ethanol), followed by its mixture with sodium methoxide (CH_3_ONa), CaN, and zinc nitrate Zn(NO_3_)_2_ dissolved in a solution of ethanol and glycol. Ammonia was used to adjust the pH to 6, and this solution was aged, dried and calcinated at 300 °C to get a glass powder [229].

#### 3.3.2. Processing and Final Formatting of Materials

HA nano powders with a controlled size have been achieved using CaN and P_2_O_5_ ethanol solutions, with a molar ratio of 10:3, and adjusting the parameters, such as the aging time and calcination temperature. A nano powder exhibiting low crystallinity, a carbonated apatitic structure, resembling that of human bone apatite with crystallites of 20–30 nm in size, was prepared through appropriate sintering at a temperature of 600 °C [230]. Likewise, HA nano powders with different sizes of 10–15, 15–25 or 50–80 nm have been monitored using, respectively, 4, 48 or 72 h of ageing [231].

Sol–gel deposition provides a convenient method for applying thin calcium phosphate (CaP) films over convoluted surfaces, such as those associated with sintered porous-surfaced dental implants. Nanocrystalline carbonated hydroxyapatite films have been prepared by dip coating using a withdrawal speed of 20 cm min^−1^ onto a porous-surfaced dental implant (Endopore^®^ implants). A precursor solution of CaN in ethanol and triethyl phosphite (firstly hydrolyzed), was aged for 2 days at room temperature. The films were further annealed at 500 °C for 20 min in air and, then, furnace cooled to room temperature [232].

Nanofibers of HA and its fluoridated form, FHA, were synthesized based on their sol–gel precursors using an electrospinning process. The fluoridation level was fixed at 25% with respect to the hydroxyl ions, by the addition of NH_4_F to the TEP solution. The fiber diameter was obtained in the range of a few micrometers to hundreds of nanometers (1.55 µm–240 nm), by means of adjusting the concentration of the sols. The FHA nanofiber produced in this study had higher chemical stability than the HA equivalent, and released fluorine efficiently following the dissolution profile [233]. A nonwoven nanofiber film made of strontium-substituted HA-CaO-CaCO_3_ nanofibers with a mesoporous structure was fabricated using the sol–gel method followed by electrospinning. CTAB was used as a porogen and poly(vinyl pyrrolidone) (PVP) and Pluronic^®^ 123 were dissolved in absolute ethanol and incorporated into the precursor solution after placing in the precursor solution and aged at 60 °C for 12 h [234].

An all-ceramic (SP1_h_HA) scaffold combining dual porosity of well-interconnected macroporous cavities and organized nanopores, as well as a HA nano-biomimetic coating, was processed by infiltration of an MBG sol and SBA-15 silica microparticles mixture slurry, which was subjected to a further 800 °C heating treatment and a final HA biomimetic coating using simulated body fluid [235]. Scanning electron microscopy (SEM) observations showed very high similarities in both the overall macrostructure and the surface microstructure between the SP1_h_HA scaffold and the commercial bone-void filler ProOsteon^®^.

#### 3.3.3. Therapeutic and Clinical Uses

Collagen infiltrated with sol–gel synthesized HA and silica nanoparticles have been proposed as suitable scaffolds for the remineralization of the dentin resulting from dental caries or acid erosion [236]. Likewise, silicon-substituted hydroxyapatite (Si-HA) materials demonstrate good potential for maxillofacial applications compared with the response to stoichiometric hydroxyapatite. A high Si content appears to promote rapid bone mineralization through in vitro osteoblasts response, since large amounts of calcium phosphate mineral started to develop across the extracellular matrix in a sample containing 5 mol% Si [237]. Sol–gel synthesized mesoporous hydroxyapatite nanoparticles exhibit excellent Vero cells cytocompatibility and viability, when loaded with methionine (MT), an essential amino acid drug, demonstrating an initial burst release followed by the slow release of the drug, which is beneficial for the speedy recovery of tissues and could be a useful material for bone tissue engineering [227].

Electrochemical impedance measurements in Ringer’s physiological solution has indicated that the development of nano HA coatings using the sol–gel method improves the corrosion resistance of implants [238]. The antibacterial properties of films made of strontium-substituted hydroxyapatite nanofibers have proven excellent drug-loading efficiency and could retard the burst release of tetracycline to maintain antibacterial activity for over 3 weeks [234]. From the perspective of dental restorative applications, (Sr/Fe) co-doped biphasic calcium phosphate dental implant coatings prepared using the sol–gel synthesis technique would be favorable for faster epithelial sealing and would also reduce the chances of infection [226].

The use of an all-ceramic scaffold, consisting of a biomimetic nano-hydroxyapatite surface coating growth onto an open and interconnected macropore structure, which also has a nano-organized porous texture, has been proposed to restore bony defects in alveolar bone. This material has been compared in vitro with a commercial control ProOsteon^®^ 500R, showing a two stage sustained release of gentamicin sulfate (GS) instead of the quick release shown by ProOsteon^®^ 500R [235].

### 3.4. Progress in Calcium Silicate Cements Research

#### 3.4.1. Compositions and Synthesis Routes

Calcium silicates, particularly tri-calcium silicate, Ca_3_SiO_5_ (C3S), and di-calcium silicate, Ca_2_SiO_4_ (C2S), compounds are very relevant in dentistry as they are the fundamental components of hydraulic cements used in endodontic procedures [239]. Both C3S and C2S react with water to form calcium silicate hydrate (CSH), which through the polymerization network contributes to the self-setting properties and increased mechanical strength after aging [240]. However, it is well reported that C2S reacts with water at a slower rate than C3S [241] and also that C2S polymorphism in their beta (β) and gamma (γ) forms have important differences, with the beta form being the most reactive [242,243].

Zhao et al. [244] reported the sol–gel synthesis of pure C3S powders after a heat treatment at 1400 °C and above, using an initial Ca/Si molar ratio of 3, from Ca(NO_3_)_2_4H_2_O (CaN) and TEOS as the precursor materials and HNO_3_ as a catalyst. The obtained C3S powder showed particles with some pores of about 1–5 µm and performed well as a self-setting workable paste, with good biocompatibility and surface bioactivity in vitro [245]. The synthesis and in vitro bioactivity of C2S compositions in their β and γ forms has also been studied using the same precursors, but modifying the Si/Ca molar ratio and the heat treatment temperatures [246,247]. More recently, the sol–gel synthesis parameters, such as the different mixing orders of reactants or the amount of nitric acid added, or the calcium silicates using a Ca/Si molar ratio of 3, has been evaluated and optimized [248]. The results from this study demonstrated that sol–gel-derived powders can be achieved showing porous microstructures and with a setting time of ~30 min, a value well below that specified for commercial silicate cements as detailed in Section 2.3.

The synthesis of endodontic cements, including Al, Zn and F, has been investigated by Voicu et al., showing an increase in the C3S crystallite size and a shifting of its XRD peaks, which suggests the presence of Zn or/and F in the C3S lattice with a positive influence on the material’s grindability [249]. An interesting material with a 15 min setting time was obtained by these authors using TEOS, aluminum butoxide (C_12_H_27_O_3_Al), zinc acetate (Zn(CH_3_COO)_2_2H_2_O) and CaN as reactive precursors and adequate thermal treatment of 1450 °C [250]. Successfully synthesized strontium-doped C3S up to Sr = 2 mol% with applicable setting times in clinical practice has been reported using TEOS, CaN and Sr(NO_3_)_2_ [251]. The synthesis of a magnesium–calcium silicate cement (Mg–CS) with Mg content of up to 10 mol% has been achieved using TEOS, CaN and Mg(NO_3_)_2_6H_2_O as precursors, nitric acid as a catalyst and absolute ethanol as the solvent, followed by heat treatment at 800 °C for 2 h and ball milling for 6 h in ethyl alcohol using a centrifugal ball mill [252]. Using similar reactive compounds, different formulations including 1, 3 or 5 mol% of Mg to satisfy the (Mg + Ca)/Si molar ratio of 3, were also investigated after heat treatment at 1400 °C. It could be seen that the Mg ion incorporated into the C3S phase and residual Mg ion remained in the Mg(OH)_2_ phase, which plays the role of hydration accelerator, and the setting time was shortened [253]. C2S, C2S–xZn and C2S–xCu powders with different percentages (x = 5% or 10%) of Zn- or Cu-substituted Ca were also synthesized by a modified sol–gel method using a silica sol (SiO_2_, containing 25.5% SiO_2_), CaN and Zn(NO_3_)_2_6H_2_O or Cu(NO_3_)_2_3H_2_O with a nominal (CaO + ZnO + CuO)/SiO_2_ molar ratio of 1.8:1, an ethanol–water mixture and heat treatment at 800 °C for 3 h [254].

#### 3.4.2. Final Processing of Materials

Workable pastes of C3S prepared by sol–gel using a liquid to powder ratio (L/P) of 0.8–1.2 mL g^−1^ were self-setting and could be injected within 15–60 min [245]. Calcium silicate cements consisting of sol–gel-derived calcium silicate powder of SiO_2_–CaO ranged from 7:3 to 3:7 were processed using a 3.7 M ammonium phosphate solution (NH_4_)_2_HPO_4_–NH_4_H_2_PO_4_ with an L/P over the range 0.5–0.7 mL g^−1^, resulting in self-hardening to form apatite and a CSH gel within 9 min [255]. Sol–gel C3S based cements were processed using ball milling mixing in combination with a 20% radiopacifier phase, such as BaZrO_3_, CaZrO_3_ and SrZrO_3,_ producing radiopaque materials, which were either comparable or else improved over the control MTA Angelus [256]. Nano powders of barium titanate (BT) and biocellulose (BC) were added (10 wt%) to calcium silicophosphate cements based on sol–gel synthesized calcium silicates, with a CaO/SiO_2_ molar ratio of 1 and an orthophosphoric acid solution partially neutralized with Al_2_O_3_ and ZnO. The addition of BT and BC nano powders determined the decrease in the setting time, whilst it did not significantly influence the mechanical properties of the resulting composites [257]. A recent paper published by our laboratory details how the use of the sol–gel route can be effective for obtaining endodontic cements with a majority of C3S content and C2S in its hydraulic beta form. Besides, the implementation of a novel post-synthesis treatment at room temperature using ethanol allows for a final product with a finer particle size and increased CaCO_3_ content, resulting in an improved material in terms of the setting time and bioactive response [258].

#### 3.4.3. Therapeutic and Clinical Uses

Bioactivity, which implies the release of calcium ions, electroconductivity and the formation of an interfacial layer between the material and dentinal wall, is a common property of calcium silicates [114]. The hydration of sol–gel synthesized C3S yields a dissolvable CSH, whose ionic products have a stimulatory effect on relevant cell growth [245]. Fast setting and controllable degrading properties, as well as the stimulation of odontogenesis/angiogenesis, has been reported for a magnesium–calcium silicate (Mg–CS) cement with a Mg content of up to 10 mol%. The Mg–CS cement has been shown to stimulate the proliferation of human periodontal ligament cells (hPDLCs) in vitro and actively promote the secretion of odontogenic (DSPP and DMP-1) and angiogenic (vWF and ang-1) proteins [252]. Also consistent with this, another study using Mg–CS with different amounts 1, 3, 5 mol% of Mg ion has been shown to promote osteogenic differentiation of human dental pulp stem cells (hDPSCs) [253].

Sol–gel synthesized C2S powders partially substituted with Zn or Cu were investigated systematically to examine their antibacterial activity in vitro and osteogenic activity in mandibular bone defects in vivo. The pure C2S cement showed a significant antibacterial response in comparison with the Zn-/Cu-substituted C2S cements in the initial several hours, but the latter could prolong the antibacterial efficacy. Also, maximum bone regeneration was consistently observed in defects filled with Zn-/Cu-substituted C2S cements [254]. 

Sol–gel synthesis of C3S including F and Zn ions in the crystal lattice has been correlated with the materials reactivity vs. water, as well as its mechanical and aesthetical properties [249]. Besides, the mixing of Sr_2_SiO_4_ particles synthesized by sol–gel to a cementing composite with C3S and C2S at a weight ratio of 30% by weight has been shown to give sufficient radio-opacity to an endodontic cement, as well as to enhance its bioactivity and tubule occlusion [259].

## 4. Conclusions

The combination of sol–gel chemistry and advances in materials processing techniques make a very promising tandem for innovation in the field of bioceramics for dentistry. The sol–gel chemical synthesis process is very versatile, allowing it: to extend the chemical composition of the materials to ranges that cannot be achieved with other more conventional processes, such as the melting of precursor compounds; to improve the textural properties of the resulting solids due to the intrinsic mesoporosity generated in the condensation process of the inorganic polymers forming the material. This mesoporosity leads to high specific surface area values and, therefore, the high surface reactivity of the resulting products; the adaptation of synthesis routes that incorporate structure-directing molecules in order to control the organization in the condensation of the inorganic material. This makes it possible to design the size of the final particles of the material in the micro and/or nanometric ranges, but also to generate ordered nanoporous structures that can significantly increase the specific surface area and adsorption of the bioceramics; the possibility of adapting the chemical synthesis process with different physical technologies in the processing of the biomaterials for their shaping into the formats that are best suited to optimize their functionality.

In relation to the toxicological and biocompatibility characteristics of sol–gel-produced bioceramics, it is important to note that they can vary depending on the specific formulations, processing conditions and intended applications. The surface properties, such as the specific surface area, nanopore size, roughness and chemistry, play a crucial role in determining the interaction between bioceramics and living tissues. Moreover, the tissue response to sol–gel products depends on various factors, including the type of tissue, implantation site and duration of exposure. As detailed in previous sections, well-designed sol–gel bioceramics have been found to promote favorable dental tissue responses, such as cell adhesion, proliferation and differentiation, as well as good biological parameters measuring dental tissue integration, inflammation, immune system response and long-term stability (see Table A1).

In terms of environmental sustainability, the sol–gel method allows for better control over the chemical composition of bioceramics minimizing material waste, as it enables precise synthesis and reduces the need for excess raw materials. It uses low reaction temperatures in comparison to other burning and high temperature thermal methods, contributing to cost savings and environmental sustainability. Furthermore, it offers ease of processing to create complex shapes and structures using simpler equipment than other high temperature or high vacuum technologies.

## Data Availability

The data presented in this study are available on request from the corresponding author.

## Sample Availability

Not available.

## Abbreviations

ICMS	Materials Science Institute of Seville
NCDs	Noncommunicable diseases
sol	Solution
BG	Bioactive glass
CaP	Calcium phosphate
CaSi	Calcium silicate
ERMI	Endosseous ridge maintenance implant
GICs	Glass-ionomer cements
MCP	Monocalcium phosphate
DCPA	Dicalcium phosphate
TCP	Tricalcium phosphate
HA	Hydroxyapatite
BECs	Bioactive endodontic cement
MTA	Mineral trioxide aggregate
GMTA	Gray ProRoot MTA
WMTA	White ProRoot MTA
TEOS	Tetraethyl orthosilicate
TEP	Triethyl phosphate
CaN	Calcium nitrate tetrahydrate
SBF	Simulated body fluid
HCA	Hydroxy carbonate apatite
MBG	Mesoporous bioactive glass
DDA	Dodecylamine
CTAB	Hexadecyltrimethyl ammonium bromide
PEG	Poly(ethylene glycol)
PEO	Poly(ethylene oxide)
PPO	Poly(propylene oxide)
P/L	Powder to liquid ratio
TMOS	Tetramethylorthosilicate
hPDLCs	Human periodontal ligament-derived cells
SP	Silica particles
SBA-15	Santa Barbara Amorphous-15
GTR	Guided tissue regeneration
TIPS	Thermally induced phase separation
GS	Gentamicin sulphate
Nf	Nanofibres
PRP	Platelet-rich plasma
MBN	Mesoporous bioactive glass nanoparticles
WSLs	White spot lesions
ALP	Alkaline phosphatase
YAG	Yttrium aluminum garnet
SA	Stearic acid
PVP	Poly(vinyl pyrrolidone)
SEM	Scanning electron microscopy
Si-HA	Silicon-substituted hydroxyapatite
MT	Methionine
C3S	Tri-calcium silicate
C2S	Di-calcium silicate
CSH	Calcium silicate hydrate
BT	Barium titanate
BC	Biocellulose
hDPSCs	Human dental pulp stem cells

## Appendix A

**Table A1:** Comparison of relevant properties for the three types of sol–gel bioceramics studied.

Bioceramic	Properties	References
BG ^1^	Highly versatile for formulating variations in chemical composition; Bioactive; Biocompatible; Osteointegrative; Osteogenic and osteoinductive; Biodegradable; Bactericide	[28,35,189,192]
CaP ^2^	Biomimetic to natural human hard tissues composition; Biocompatible; Osteoconductive; Bioresorbable	[88,89,227,232]
CaSi ^3^	Hydraulic; Biocompatible; Bioactive; Osteoconductive; Osteoinductive	[66,115,156,258]

^1^ Bioactive glass; ^2^ calcium phosphate; ^3^ calcium silicate.
